# New ambuic acid derivatives from the solid culture of *Pestalotiopsis neglecta* and their nitric oxide inhibitory activity

**DOI:** 10.1038/srep09958

**Published:** 2015-05-19

**Authors:** Qiu-Yue Qi, Er-Wei Li, Jun-Jie Han, Yun-Fei Pei, Ke Ma, Li Bao, Ying Huang, Feng Zhao, Hong-Wei Liu

**Affiliations:** 1 State Key Laboratory of Mycology, Institute of Microbiology, Chinese Academy of Sciences, NO. 1 Beichen West Road, Chaoyang District, Beijing 100101, People’s Republic of China; 2 University of Chinese Academy of Sciences, No.19A Yuquan Road, Beijing 100049, People’s Republic of China; 3 State Key Laboratory of Microbial Resources, Institute of Microbiology, Chinese Academy of Sciences, NO. 1 Beichen West Road, Chaoyang District, Beijing 100101, People’s Republic of China; 4 School of Pharmacy, Yantai University, No. 32 Qingquan Road, Laishan District, Yantai, 264005, People’s Republic of China

## Abstract

Four new ambuic acid derivatives (1–4), and four known derivatives (5–8), were isolated from the solid culture of a plant pathogenic fungus *Pestalotiopsis neglecta*. Their structures were elucidated by extensive NMR experiments. The absolute configuration of the C-16 secondary alcohol in 1 was deduced via the CD data of the in situ formed [Rh_2_(OCOCF_3_)_4_] complex with the acetonide derivative of 1. The absolute configuration in 3 was assigned by comparison of the experimental and simulated electronic circular dichroism (ECD) spectrum. The NMR data of compound 5 was reported for the first time. In the nitric oxide (NO) inhibition assay, compounds 4, 6 and 7 showed inhibitory activity against the NO production in the lipopolysaccharide (LPS)-induced macrophage with IC_50_ values of 88.66, 11.20, and 20.80 µM, respectively.

Nitric oxide (NO) produced by a group of nitric oxide synthases (NOSs) is highly diffusible across cell membranes and modifies many biological molecules^1^,^2^. Overproduction of NO is proved to be closely related with many pathogenic diseases, such as inflammation and cancer^3^. Through reducing NO formation or scavenging NO molecule, NO inhibitors may be used as powerful therapeutic agents^4^.

The fungi belonging to the genus of *Pestalotiopsis* have attracted much attention due to their ability in producing diverse secondary metabolites with various biological activities^5^,^6^,^7^,^8^,^9^,^10^. The anticancer drug taxol has been reported from the culture of several species of *Pestalotiopsis*, which provides an alternative way for the production of this valuable drug^11^,^12^. In our searching for natural NO inhibitors from fungi, the pathogenic fungus *P. neglecta* (FJ–2) was separated from the twig of *Camellia sinensis* growing in Fujian Province of China. The fungus was grown in a solid-substrate fermentation culture. The EtOAc extract of its solid culture was found to have NO inhibitory activity (IC_50_ = 250 µg/mL). Chemical investigation on its EtOAc extract afforded four new ambuic acid derivatives (**1**–**4**), and four known compounds^13^,^14^ **5**–**8**. Details of the isolation, structure elucidation, and NO inhibitory activity of these compounds are reported herein.

## Results and Discussion

The ethyl acetate extract of the solid culture of the fungus *P. neglecta* was isolated by silica gel column chromatography and ODS column chromatography, and finally purified through reversed-phase high performance liquid chromatography (HPLC) to give eight ambuic acid derivatives (**1**–**8**). The structures of known compounds (**6**–**8**) were determined by NMR data analyses and comparison with the literature data^13^,^14^.

**Figure 1:**
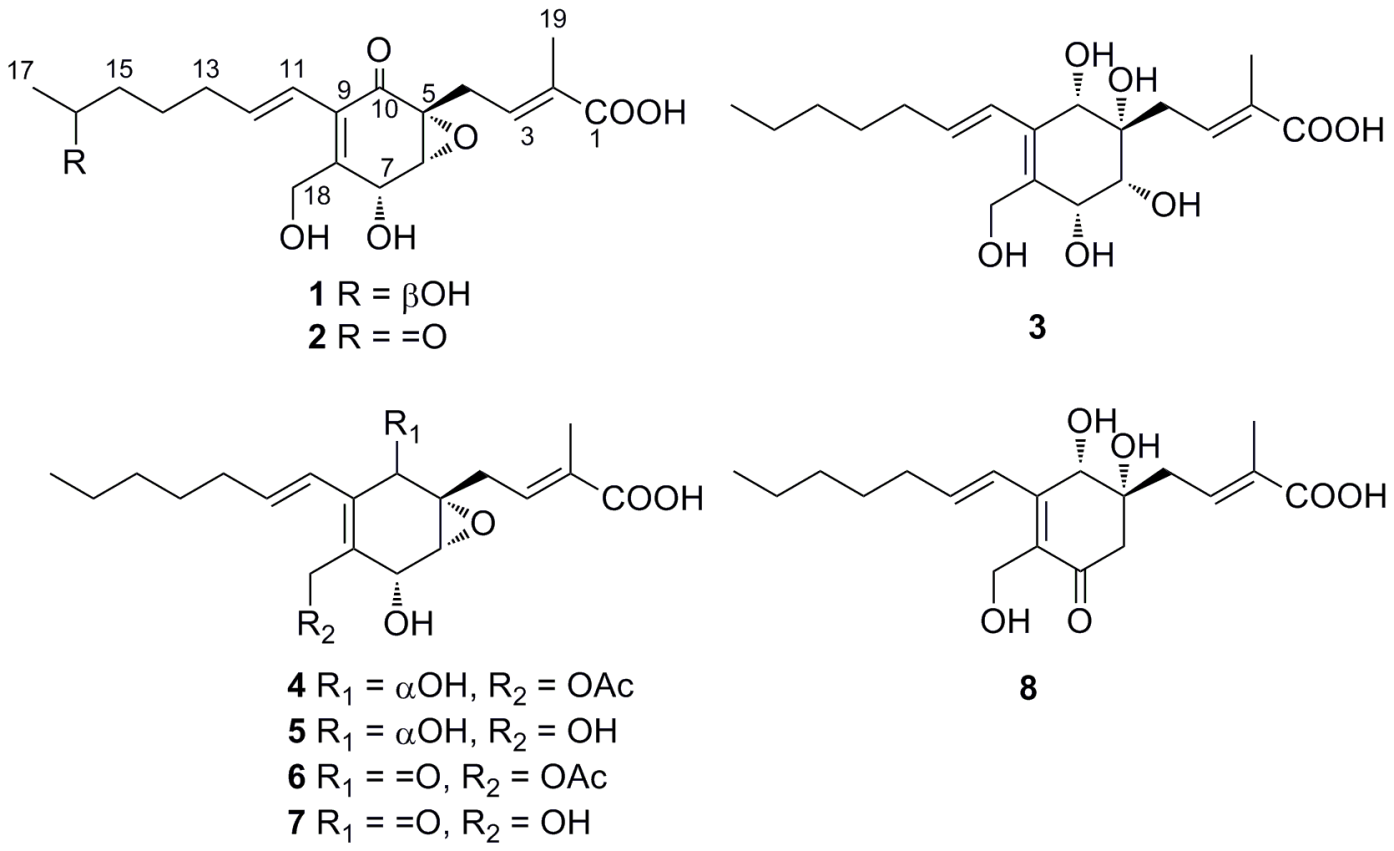
Chemical structures of compounds 1–8.

**Figure 2:**
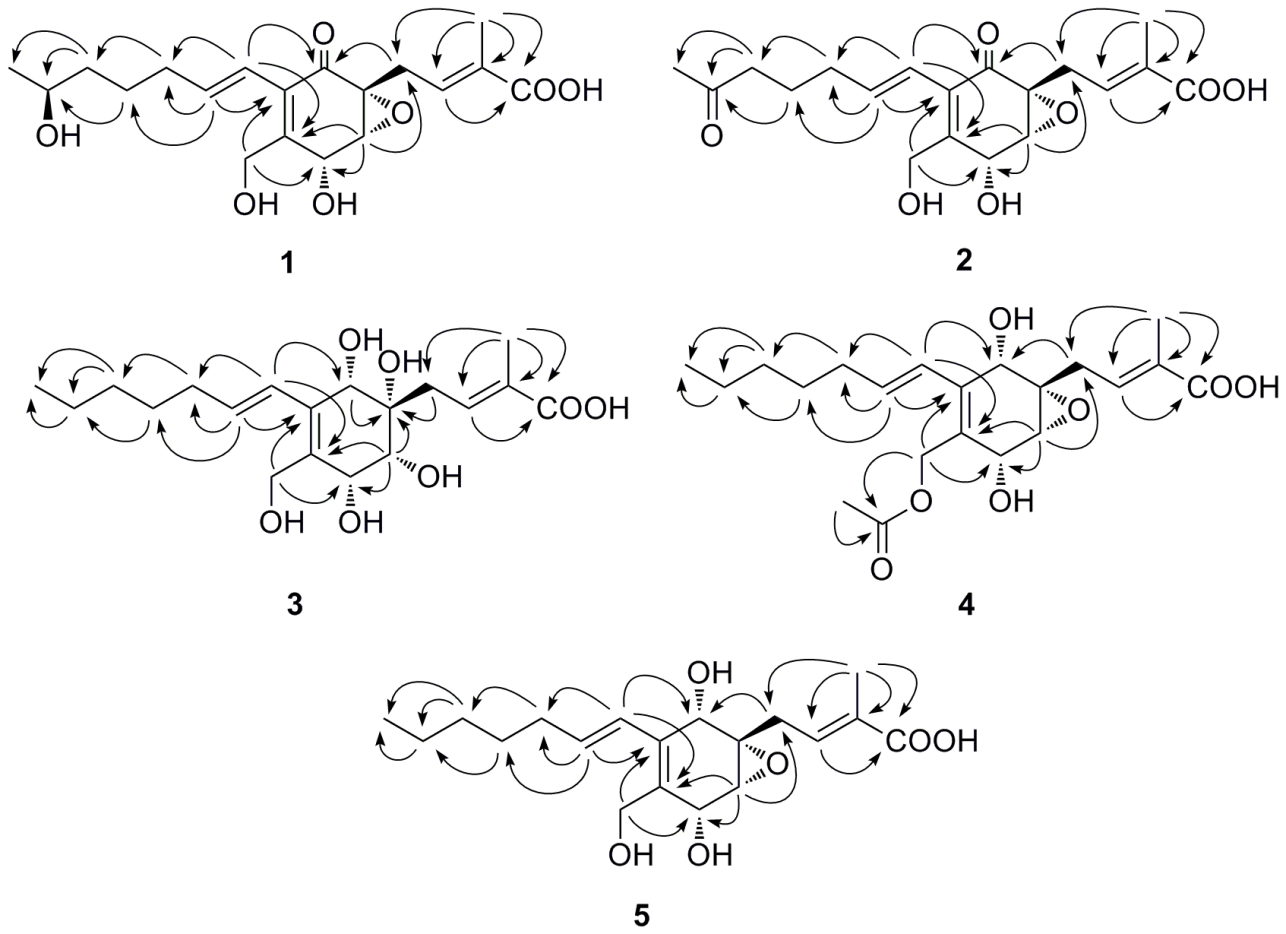
Key HMBC 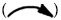 correlations of compounds 1–5.

**Figure 3:**
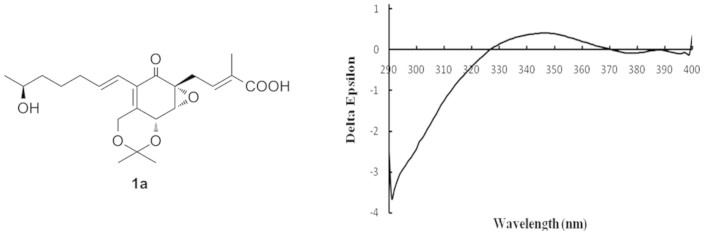
CD spectrum of Rh-complex of 1a with the inherent contributions subtracted.

Compound **1** (Figure 1) was isolated as colorless oil. It was assigned the molecular formula C_19_H_26_O_7_ (seven degrees of unsaturation) on the basis of HRESIMS analysis (*m/z* = 389.1570 [M + Na]^+^). Its^1^H and ^13^C NMR spectra showed resonances for two methyl groups, five methylenes (one oxygenated), three *O*-methines, six olefinic carbons (three of which were protonated), one oxygenated sp^3^ quaternary carbon, one carboxylic carbon (*δ*_C_ 171.1, C-1), one α, β-unsaturated ketone carbon (*δ*_C_ 196.0, C-10). These data (Table 1) accounted for all the NMR resonances for **1**. The^1^H-^1^H COSY NMR data of **1** showed the three isolated spin-systems of C-4–C-3–C-19 (allylic coupling between H-3 with H_3_-19), C-6–C-7 and C-11–C-17. HMBC correlations from H_2_-4 to C-5, C-6, and C-10 (*δ*_C_ 196.0), H-6 to C-5, C-7, and C-8 (*δ*_C_ 150.8), and from H_2_-18 to C-7, C-8 and C-9 (*δ*_C_ 131.9 ), H-11 to C-8, C-9 and C-10 (Figure 2) established the C-5–C-10 cyclohex-2-en-one moiety with the C-4, C-18 hydroxymethyl unit [*δ*_H_ 4.41 d (*J* = 12.9 Hz), H-18a; *δ*_H_ 4.52 d (*J* = 12.9 Hz), H-18b; *δ*_C_ 60.3, C-18] and C-11 attached to C-5, C-8 and C-9, respectively. HMBC correlations from H-3 to C-1 (*δ*_C_ 171.1), C-19, from H_3_-19 to C-1, C-2 and C-3 suggested H_3_-19 methyl and carboxylic group were both located at C-2. One hydroxy group was located at C-16 [*δ*_H_ 3.73 q (*J* = 6.2 Hz); *δ*_C_ 68.4, C-16] by correlations from H_3_-17, H_2_-14 and H_2_-15 to C-16. Considering the chemical shifts of C-5 (*δ*_C_ 61.2; 61.3 in ambuic acid^13^), C-6 (*δ*_C_ 61.1; 61.1 in ambuic acid^13^), and C-7 (*δ*_C_ 65.9; 66.0 in ambuic acid^13^) and the unsaturation requirement of **1**, the remaining one unsaturation degree was due to an epoxide ring at C-5 and C-6 of the cyclohex-2-en-one. This was furthermore confirmed by the acetonide **1a** (Figure 3) that was yielded from the addition of an acetone unit with the hydroxyl groups at C-7 and C-18. On the basis of these data, the planar structure of **1** was proposed.

The relative configurations of **1** were deduced by the^1^H-^1^H coupling constants and NOESY data. The C-11/C-12, C-2/C-3 double bonds were assigned *E*-geometry on the basis of the large coupling constant (*J* = 15.9 Hz) observed between H-11 and H-12, and NOESY correlation of H_2_-4 with H_3_-19. The small vicinal coupling constant (*J*_6,7_ = 2.8 Hz) suggested a *cis* orient between H-6 and H-7, the NOESY correlation of H-6 with H_2_-4 indicated these protons were on the same face of the cyclohex-2-en-one ring. The CD spectrum of **1** showed a positive (350 nm) and a negative (240 nm) Cotton effects, which were similar to those of macrophorin A^15^, (+)-epoxydon^16^, and ambuic acid derivatives^14^, suggesting the *5R*, *6R*, and *7R* absolute configuration for **1**. The absolute configuration of the C-16 secondary alcohol in **1** was deduced via the CD data of the in situ formed [Rh_2_(OCOCF_3_)_4_] complex with acetonide **1a** (Figure 3). The sign of the E band (at ca. 350 nm) can be used to correlate the absolute configuration of a secondary alcohol by applying the bulkiness rule^17^,^18^. The Rh complex of **1a** displayed a positive Cotton effects at near 350 nm, suggesting the 16*S* absolute configuration^17^.

Compound **2** (Figure 1) gave a pseudomolecular ion [M + Na]^+^ peak at *m/z* 387.1414 by HRESIMS, consistent with the molecular formula C_19_H_24_O_7_ (eight degrees of unsaturation). Its ^1^H and ^13^C NMR spectroscopic data (Table 1)revealed structural similarity to **1**, except that one oxygenated methylene [*δ*_H_ 3.73 q (*J* = 6.2 Hz); *δ*_C_ 68.4, C-16] in **1** was replaced by a ketone (*δ*_C_ 211.8, C-16). It was confirmed by HMBC correlations from H_3_-17, H_2_-14 and H_2_-15 to C-16 (Figure 2). Comparison of the ^1^H-^1^H coupling constants and NOESY data of **2** with those of **1** indicated that both compounds possess the same relative configurations at C-5, C-6, C-7, and C-11/C-12, C-2/C-3 double bonds. The absolute configuration of C-5, C-6 and C-7 in **2** was deduced on the basis of CD data. The CD spectrum of **2** (Figure S6) showed a positive (350 nm) and a negative (240 nm) Cotton effects, correlating to the *5R*, *6R*, and *7R* configuration.

Compound **3** (Figure 2) was assigned the molecular formula C_19_H_30_O_7_ (five degrees of unsaturation) by HRESIMS (*m/z* = 393.1887 [M + Na]^+^). Analysis of its NMR data (Table 1) revealed that **3** possess the similar structural feature to ambuic acid (**7**), except that the carbonyl group at C-10 in **7** was replaced by an oxygenated methine in **3**. Such variation was confirmed by HMBC cross peaks from H-10 to C-4, C-5, C-6, C-8, C-9, and C-11 (Figure 2). The chemical shifts of C-5 (*δ*_C_ 74.2), C-6 (*δ*_C_ 74.0), C-7 (*δ*_C_ 72.5), and C-10 (*δ*_C_ 72.4), the molecular weight of **3**, as well as the requirement of unsaturation degree of **3** indicated the opening of the epoxide ring at C-5 and C-6 in **1** and the substitution of hydroxyl group at C-5, C-6, C-7, and C-10. The small vicinal coupling constant (*J*_6,7_ = 2.3 Hz) suggested a *cis* orient between H-6 and H-7, the NOESY correlation of H-6 with H-4b [*δ*_H_ 2.91 dd (*J* = 15.1, 6.8 Hz)], and H-10 with H-4a [*δ*_H_ 2.70 dd (*J* = 15.1, 6.8 Hz)] indicated H_2_-4, H-6, H-7, and H-10 were on the same face of the cyclohex-2-en-one ring.

**Figure 4:**
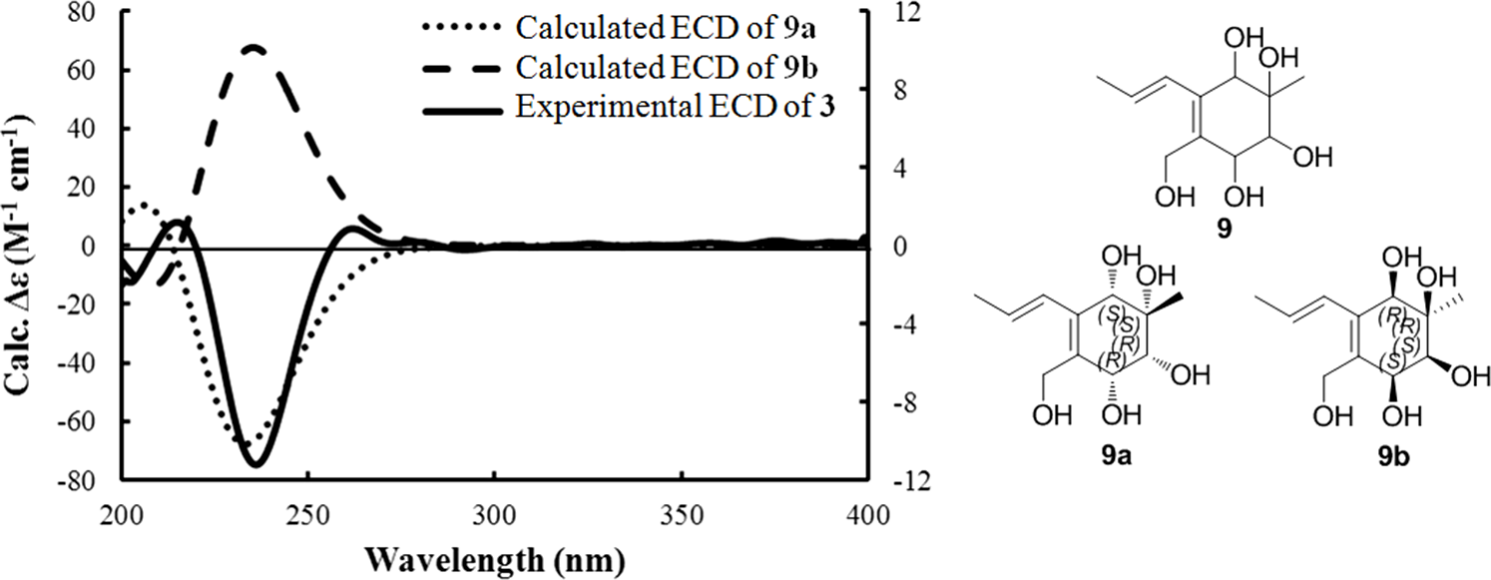
The experimental CD spectrum of 3 in methanol and the calculated ECD spectra of 9a and 9b. Structures 9a and 9b represent two possible stereoisomers of 9.

Since the cyclohex-2-en-one ring system in **3** was relatively rigid, which would significantly affect the CD property, whereas the conformationally flexible side chain had insignificant effect on the CD spectrum of **3**, a simplified structure **9** was used for ECD calculations (Figure 4). Considering the relative configuration determined above, one of the two enantiomers (*5S*, *6R*, *7R*, *10S*)-**9a**, (*5R*, *6S*, *7S*, *10R*)-**9b** should represent the actual absolute configuration of **9**. The MMFF94 conformational search followed by B3LYP/6-31G(d) DFT reoptimization afforded four lowest-energy conformers for enantiomers **9a** and **9b**, respectively (Figure S10). The calculated ECD spectra of enantiomers **9a** and **9b** were then generated by Boltzmann weighting of the conformers (Figure 4). The absolute configuration of **3** was deduced by comparison of the experimental and simulated electronic circular dichroism (ECD) curves of **9a** and **9b**. The experimental CD spectrum of **3** was comparable only to the calculated ECD curve of **3a**. Therefore, the absolute configuration of **3** was deduced to be *5S*, *6R*, *7R*, *10S*.

Compound **4** (Figure 1) gave a pseudomolecular ion [M + Na]^+^ peak at *m/z* 417.1881 by HRESIMS, consistent with the molecular formula C_21_H_30_O_7_ (seven degrees of unsaturation). Analysis of its NMR data (Table 2) revealed that **4** possess the similar structure to **1**, except that C-10 ketone in **1** was reduced to a hydroxyl, the C-16 hydroxyl group was replaced by the hydrogen, and the C-18 hydroxy was acetylated. These observations were supported by HMBC cross-peaks from H-10 to C-4, C-5, C-8, C-9, H_3_-17 to C-15, C-16, and from H_2_-18, H_3_-21 to C-20 (Figure 2). The relative configurations for C-5, C-6, and C-7 in **4** were deduced to be the same as those in **1** by comparison of the ^1^H-^1^H coupling constants and NOESY data for relevant protons. In 1D NOE experiment of **4**, upon irradiation of H-10, enhancements were observed for H_2_-4 and H-6, suggesting that H-10, H_2_-4, and H-6 were on the same face of the cyclohex-2-en-one ring. The CD spectrum of **4** (Figure S13) was nearly identical to that of **3**, both showing significant negative Cotton effects (CEs) in the regions of 220–260 nm. Therefore, **4** was deduced to have the 5*S*, 6*R*, 7*R*, 10*S* absolute configuration.

Compound **5** (Figure 1) gave a pseudomolecular ion [M + Na]^+^ peak at *m/z* 375.1780 by HRESIMS, consistent with the molecular formula C_19_H_28_O_6_ (six degrees of unsaturation). Analysis of its NMR data (Table 2) revealed that **5** possess the similar structure to **4**, except that C-18 *O*-acetyl group was missed. The relative configurations of **5** were deduced to be the same as those in **4** by comparison of the ^1^H-^1^H coupling constants and NOESY data for relevant protons. The CD spectrum of **5** (Figure S16) was matched to those of **3** and **4**, both showing significant negative Cotton effects (CEs) in the regions of 220–260 nm. Therefore, compound **5** was deduced to have the 5*S*, 6*R*, 7*R*, 10*S* absolute configuration. Although previously included in the databases of SciFinder Scholar and PubChem, the information about its isolation and structure assignment was not published. Herein, we report the isolation and NMR data of **5** for the first time.

Ambuic acid (**7**) and its derivatives have been isolated from the plant endophytic fungus *Pestalotiopsis* sp. and *Monochaetia* sp., and from the endolichenic fungus *Pestalotiopsis* sp. Ambuic acid showed moderate antifungal effects against several plant pathogenic fungi *Fusarium solani*, *Fusarium cubense*, *Helminthosporium sativum*, *Diplodia natelensis*, *Cephalosporium gramineum*, *Pythium ultimum*^13^, and antimicrobial against the Gram-positive bacterium *Staphyococcus aureus*^14^. Ambuic acid also inhibits the biosynthesis of cyclic peptide quormones in Gram-positive bacteria *Enterococcus faecalis*^19^. In this study, compounds **2**–**8** were evaluated for their antifungal activity against other three different pathogenic fungi *Aspergillus fumigatus*, *Aspergillus flavus*, and *Fusarium nivale*. They did not show any antifungal activity against tested strains at the concentration of 200 µg/mL.

Macrophages play major roles in inflammation and host defense mechanisms against bacterial and viral infections^20^. The NO radical produced by the oxidation of L-arginine by NO synthase (NOS) is an effective molecule for the anti-inflammatory and anti-microbial effects of macrophages. However, excessive production of NO may lead to severe injury to host cells and tissues during acute and chronic inflammation. To confirm the bioactive secondary metabolites responsible for the NO inhibitory activity detected in the culture extract of *P. neglecta*, compounds **1**, **4**–**8** were tested for their inhibitory activity against the NO production in LPS-induced macrophages (Table 3), Compounds **2** and **3** were obtained with fewer amounts (≤1.0 mg) and not enough to test their NO inhibitory activity. In comparison with the positive control of hydrocortisone (IC_50_ = 53.68 µM), compounds **6** and **7** showed strong NO inhibitory activity with IC_50_ of 11.20 and 20.80 µM, and compound **4** showed weak inhibitory activity with IC_50_ of 88.66 µM. All other tested compounds exhibited marginal NO inhibitory activity with IC_50_ larger than 100 µM. It can be presumed that the major secondary metabolites ambuic acid derivative (**6**) contribute mainly to the NO inhibitory activity detected in the culture. *In vivo* experiments for **6** are under test. The further studies of anti-inflammatory test for other minor secondary metabolites and more advanced structure-activity relationship of these analogues were not continued due to fewer amounts of these compounds.

In conclusion, eight ambuic acid derivatives including four new secondary metabolites (**1**–**4**) were isolated from the culture of the pathogenic fungus *P. neglecta*. Known compounds **6** and **7** presented strong NO inhibition, which makes them be potential anti-inflammatory agents. The in *vivo* anti-inflammation assay and action mechanism deserve further investigation.

## Methods

### General experimental procedures

The optical rotations were measured on a Perkin-Elmer 241 polarimeter (Waltham, USA) and UV spectra were determined on a Thermo Genesys-10S UV-Vis spectrophotometer (Madison, USA). IR data were measured using a Nicolet IS5FT-IR spectrophotometer (Madison, USA). CD spectra were recorded on a JASCO J-815 Spectropolarimeter (Tokyo, Japan). ^1^H and ^13^C NMR data were acquired with a Bruker Avance-500 spectrometer (Rheinstetten, Germany) using solvent signals (methanol-*d*_4_, *δ*_H_ 3.30/*δ*_C_ 49.9; deuterated chloroform, *δ*_H_ 7.26/*δ*_C_ 77.7) as references. The HMQC and HMBC experiments were optimized for 145.0 and 8.0 Hz, respectively. HR-ESI-MS data were acquired using an Agilent Accurate-Mass-Q-TOF LC/MS 6520 instrument (Santa Clara, USA). TLC was carried out on Silica gel HSGF254 and the spots were visualized by spraying with 10% H_2_SO_4_ and heating. Silica gel (Qingdao Haiyang Chemical Co., Ltd., Qingdao, People’s Republic of China), and ODS (Lobar, 40–63 µm, Merck, Darmstadt, Germany) were used for column chromatography. Preparative HPLC was performed on an Agilent 1200 HPLC system (Santa Clara, USA) using an ODS column (RP-18, 250 × 10 mm, YMC Pack, 5 μm; detector: UV; Kyoto, Japan) with a flow rate of 2 mL/min. Solvents used for extraction and chromatographic separation were analytical grade.

### Fungal material

The pathogenic strain (FJ-2) used in this work was isolated from the twig of *Camellia sinensis* growing in Fujian Province of China. The fungus was identified on the basis of the DNA sequences of the ITS1-5.8S-ITS2, ITS regions of their ribosomal RNA gene. The sequence data derived from the fungal strain has been submitted and deposited at GenBank with accession number KJ719299. BLAST search result showed that the sequence was similar (99%) to the sequence of *Pestalotiopsis neglecta* (Thüm.) Steyaert in GenBank with the accession number of JX854541.

The fungal strain was cultured on slants of potato dextrose agar at 25°C for 10 d. Agar plugs were inoculated in 500 mL Erlenmeyer flask containing 120 mL of media (0.4% glucose, 1% malt extract, and 0.4% yeast extract, the final pH of the medium was adjusted to 6.5 before sterilization), and incubated at 25°C on a rotary shaker at 180 rpm for 4 d. Large scale fermentation was carried out in 40 bottles of 500 mL Fernbach flasks each containing 80 g of rice and 100 mL of distilled water. Each flask was inoculated with 10.0 mL of culture medium and incubated at 25°C for 40 d.

### Extraction and isolation

The fermented rice substrate was extracted thoroughly with ethyl acetate (3 × 5 L), and the organic solvent was concentrated under reduced pressure to afford the crude extract (15.0 g), which was subjected to a silica gel column chromatography (CC) with a gradient of n-hexane-ethyl acetate, dichloromethane-methanol to provide five fractions (A-E).

Fraction C (2.4 g) eluted with dichloromethane-methanol (20:1, v/v) was subjected to ODS CC eluting with a gradient of methanol in water (10%, 20%, 30%, 40%, 50%, 60%, 70%, 80%, 90%, 100%) to give twenty-one subfractions (C1–C21). Compound **2** (1.0 mg, *t*_R_ 62.0 min) was purified from subfraction C4 (35 mg) by RP-HPLC using 18% acetontrile in water. Compound **4** (6.9 mg, *t*_R_ 112.0 min) was obtained from subfraction C10 (87 mg) by RP-HPLC using 30% acetontrile in water. Subfraction C13 (170 mg) was separated by RP-HPLC using 63% methanol in water to afford compound **7** (25.2 mg, *t*_R_ 37.7 min). Further purification of subfraction C17 (74 mg) on RP-HPLC using 45% acetontrile in water gave compound **6** (4.5 mg, *t*_R_ 35.0 min).

Fraction D (2.0 g) eluted with dichloromethane-methanol (10:1, v/v) was separated by ODS CC eluted with a gradient of methanol in water (25%, 30%, 40%, 50%, 60%, 70%, 80%, 90%, 100%) into twenty-seven subfractions (D1–D27). Further purification of subfraction D5 (50 mg) on RP-HPLC using 30% acetontrile in water gave compound **1** (9.5 mg, *t*_R_ 6.3 min), and compound **8** (3.5 mg, *t*_R_ 20.5 min). Compound **3** (0.6 mg, *t*_R_ 20.8 min) was obtained from subfraction D7 (10 mg) by RP-HPLC using 30% acetontrile in water. Compound **5** (47.5 mg, *t*_R_ 22.3 min) was purified from subfraction D10 (81 mg) by RP-HPLC using 33% acetontrile in water.

### Absolute conguration of the secondary alcohol in 1

In order to determine the absolute configuration of the secondary alcohol in **1**, the 7,18-diol in **1** was protected by the derivatization of acetone fork/cross^21^. Compound **1** (2.0 mg) was treated with 2,2-dimethyoxypropane (1 mL) and pyridinium *p*-toluene sulfonate (1 mg), then stirred at 30°C for about 8 h under N_2_ circumstance. The reaction solution was evaporated under reduced pressure and purified by RP-HPLC using 30% acetontrile in water for 8 min following 35% acetontrile in water for 16 min to yield the acetonide **1a** (1.0 mg).

A sample of **1a** (0.5 mg) was dissolved in a dry solution of the stock [Rh_2_(OCOCF_3_)_4_] complex (1.5 mg) in CH_2_Cl_2_ (300 µL) and was subjected to CD measurements. The first CD spectrum was recorded immediately after mixing and its time evolution was monitored until stationary (ca. 10 min after mixing). The inherent CD was subtracted. The observed sign of the E band at around 350 nm in the induced CD spectrum was correlated to the absolute configuration of the C-16 secondary alcohol moiety^17^,^18^.

### Computational details

Molecular Operating Environment (MOE) ver. 2009.10. (Chemical Computing Group, Canada) software package was used to systematic conformational analyses for compound **9** with the MMFF94 molecular mechanics force field. The MMFF94 conformational analysis was optimized using DFT at B3LYP/6-31G(d) basis set level by Gaussian03 package. The 30 lowest electronic transitions were calculated using the TDDFT methodology at the B3LYP/6-31G(d) level. ECD spectra were stimulated using a Gaussian function with a half-bandwidth of 0.34 eV. The overall ECD spectra were then generated according to Boltzmann weighting of each conformer. The systematic errors in the prediction of the wavelength and excited-state energies are compensated for by employing UV correlation.

### Inhibition of NO production by the activated macrophage-like cell line RAW 264.7

Mouse monocyte-macrophages RAW 264.7 (ATCC TIB-71) were purchased from the Chinese Academy of Science. RPMI 1640 medium, penicillin, streptomycin, and fetal bovine serum were purchased from Invitrogen (NewYork, USA). Lipopolysaccharide (LPS), DMSO, 3-(4,5-dimethylthiazol-2-yl)-2,5-diphenyl-2H-tetrazolium bromide (MTT), and hydrocortisone were obtained from Sigma Chemical Co. (Saint Louis, MO, U.S.A). RAW 264.7 cells were kept in RPMI 1640 medium supplemented with penicillin (100 U/mL), streptomycin (100 μg/mL), and 10% heat-inactivated fetal bovine serum at 37°C in a humidified incubator with 5% CO_2_ and 95% air. RAW 264.7 cells were passaged by trypsinization until they attained confluence and were used for assays during the exponential growth phase.

Compounds **1**, **4**–**8** were dissolved in DMSO and were further diluted with the culture medium to give a final DMSO concentration of 0.2% in the assay. This concentration of DMSO had no significant effect on the growth of the cell line tested. The procedure of NO inhibition assay was conducted as previously reported. The level of NO was assessed by measuring the accumulation of nitrite (NO_2_^−^) using the same method as previously reported^22^,^23^. Cytotoxicity was measured using the 3-(4,5-dimethylthiazol-2-yl)-2.5-diphenyltetrazolium bromide (MTT) assay method. The inhibition rate was calculated based on the following equation (1).

equation (1):
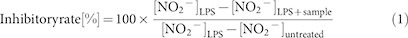
Antifungal assay

Antifungal assay was conducted in triplicate according to the National Center for Clinical Laboratory Standards (NCCLS) recommendations. The fungi were obtained from China General Microbial Culture Collection (CGMCC) and the American Type Culture Collection (ATCC), and grown on potato dextrose agar (PDA). The fungal were grown on broth cultures that were incubated at 28°C for 48 h, and the final suspensions contained 1 × 10^4^ hyphae/mL. Test samples (10 mg/mL at stock solution in DMSO and serial dilutions) were transferred to 96-well plates in triplicate, and 200 ul suspensions of fungal were added to each well with alamar blue (10 μL of 10% solution) as indicator. After incubation at 28°C for 48 h, the fluorescence intensity was measured at Ex/Em = 544/590 nm using a microtiter plate reader. The inhibition rate and IC_50_ were calculated.

compound **1**: colorless oil (methanol); [*α*]25 D 106.9 (*c* 0.16, methanol); UV (methanol) *λ*_max_ (log *ε*): 217 (3.84), 230 (3.89), 273 (3.58) nm; CD (*c* 9.8 × 10-4 M, methanol): *λ* (Δ*ε*) 219 (5.20), 241 (−0.43), 270 (2.68), 318 (−0.18), 348 (0.11); IR (neat) *v*_max_: 3369, 2965, 2931, 2858, 1682, 1435, 1381, 1264, 1205 cm^−1^; for ^1^H NMR and ^13^C NMR data see Table 1; Positive HR-ESI-MS: m/z 389.1570 [calcd. for C_19_H_26_O_7_Na (M + Na)^+^, 389.1571].

compound **2**: colorless oil (methanol); [*α*]25 D 111.0 (*c* 0.1, methanol); UV (methanol) *λ*_max_ (log *ε*): 217 (3.85), 230 (3.87), 270 (3.58) nm; CD (*c* 6.9 × 10-4 M, methanol): *λ* (Δ*ε*) 218 (7.03), 240 (−1.04), 270 (3.41), 316 (−0.19), 350 (0.29); IR (neat) *v*_max_: 3368, 2959, 2923, 2852, 1685, 1438, 1379, 1261, 1207 cm^−1^; for ^1^H NMR and ^13^C NMR data see Table 1; Positive HR-ESI-MS: m/z 387.1414 [calcd. for C_19_H_24_O_7_Na (M + Na)^+^, 387.1414).

compound **3**: colorless oil (methanol); [*α*]25 D 15.0 (*c* 0.02, methanol); UV (methanol) *λ*_max_ (log *ε*): 230 (3.62) nm; CD (*c* 2.7 × 10-4 M, methanol): *λ* (Δ*ε*) 215 (1.21), 236 (−11.21), 262 (0.88); IR (neat) *v*_max_: 3350, 2956, 2926, 2854, 1739, 1685, 1412, 1260, 1207 cm^−1^; for ^1^H NMR and ^13^C NMR data see Table 1; Positive HR-ESI-MS: m/z 393.1887 [calcd. for C_19_H_30_O_7_Na (M + Na)^+^, 393.1884].

compound **4**: brown oil (methanol); [*α*]25 D 3.6 (*c* 0.23, methanol); UV (methanol) *λ*_max_ (log *ε*): 230 (4.41) nm; CD (*c* 6.2 × 10-4 M, methanol): *λ* (Δ*ε*) 216 (0.17), 236 (−1.80), 259 (0.28), 271 (−0.05), 301 (0.07), 347 (−0.13); IR (neat) *v*_max_: 3391, 2959, 2931, 2859, 1697, 1653, 1434, 1380, 1204 cm^−1^; for ^1^H NMR and ^13^C NMR data see Table 2; Positive HR-ESI-MS: m/z 417.1881 [calcd. for C_21_H_30_O_7_Na (M + Na)^+^, 417.1884].

compound **5**: brown oil (methanol); [*α*]25 D 20.5 (*c* 0.22, methanol); UV (methanol) *λ*_max_ (log *ε*): 230 (4.21), 283 (3.43) nm; CD (*c* 1.4 × 10-3 M, methanol): *λ* (Δ*ε*) 213 (1.37), 238 (−1.72), 265 (0.72), 271 (−0.05), 301 (0.07), 347 (−0.13); IR (neat) *v*_max_: 3377, 2957, 2932, 2859, 1691, 1654, 1458, 1420, 1204 cm^−1^; for ^1^H NMR and ^13^C NMR data see Table 2; Positive HR-ESI-MS: m/z 375.1780 [calcd. for C_19_H_28_O_6_Na (M + Na)^+^, 375.1778].

**1a** ^1^H NMR (500MHz, CDCl_3_): *δ* 6.74 (1H, t, *J* = 7.5 Hz, H-3), 5.92 (1H, overlap, H-11), 5.92 (1H, overlap, H-12), 4.79 (1H, br s, H-7), 4.54 (2H, s, H-18), 3.80 (1H, q, *J* = 6.1 Hz, H-16), 3.67 (1H, d, *J* = 2.1 Hz, H-6), 2.98 (1H, dd, *J* = 16.0, 7.5 Hz, H-4b), 2.77 (1H, dd, *J* = 16.0, 7.5 Hz, H-4a), 2.18 (2H, m, H-13), 1.89 (3H, s, H-19), 1.55 (2H, m, H-14), 1.49 (3H, s, H-21), 1.46 (2H, m, H-15), 1.46 (3H, s, H-22), 1.19 (3H, d, *J* = 6.1 Hz, H-17). ^13^C NMR (125MHz, CDCl_3_): 193.7 (C, C-10), 170.5 (C, C-1), 147.1 (C, C-8), 138.7 (CH, C-12), 136.4 (CH, C-3), 130.6 (C, C-2), 125.5 (C, C-9), 120.2 (CH, C-11), 101.2 (C, C-20), 68.1 (CH, C-16), 65.1 (CH, C-7), 60.9 (CH_2_, C-18), 59.5 (C, C-5), 55.6 (CH, C-6), 38.8 (CH_2_, C-15), 33.8 (CH_2_, C-13), 27.6 (CH_2_, C-4), 25.4 (CH_2_, C-14), 24.3 (CH_3_, C-21), 24.1 (CH_3_, C-22), 23.7 (CH_3_, C-17), 12.7 (CH_3_, C-19). HMBC (500 MHz, CDCl_3_): H-3 → C-1, C-4, C-5, C-19; H_2_-4 → C-2, C-3, C-5, C-6; H-6 → C-5, C-7, C-8; H-7 → C-8, C-9; H-11 → C-8, C-9, C-10, C-12, C-13; H-12 → C-9, C-11, C-13; H_2_-13 → C-11, C-12, C-14, C-15; H_2_-14 → C-13, C-15, C-16; H_2_-15 → C-13, C-14, C-16, C-17; H-16 → C-17; H_3_-17 → C-15, C-16; H_2_-18 → C-7, C-8, C-9, C-20; H_3_-19 → C-1, C-2, C-3; H_3_- 21→ C-20, C-22; H_3_-22 → C-20, C-21. Positive HR-ESI-MS: m/z 429.1886 [calcd. for C_22_H_30_O_7_Na (M + Na)^+^, 429.1884].

## Author Contributions

L.B. and H.W.L. designed experiments. Q.Y.Q. and E.W.L. performed the isolation of compounds, and analyzed N.M.R. and M.S. data. J.J.H., Y.F.P., K.M., Y.H. and F.Z. conducted experiments. The manuscript was prepared by Q.Y.Q., E.W.L., L.B. and H.W.L. All authors reviewed the manuscript.

## Additional Information

**How to cite this article**: Qi,Q.-Y. *et al.* New ambuic acid derivatives from the solid culture of Pestalotiopsis neglecta and their nitric oxide inhibitory activity. *Sci. Rep.* **5**, 9958; doi: 10.1038/srep09958 (2015).

## Supplementary Material

Supplementary Information Supplementary information

## Figures and Tables

**Table 1:** ^1^H and 13C NMR spectroscopic data of compounds **1–3** in CD_3_OD^a^

No.	1		2		3	
	*δ*_C_	*δ*_H_ (mult., *J* in Hz)	*δ*_C_	*δ*_H_ (mult., *J* in Hz)	*δ*_C_	*δ*_H_ (mult., *J* in Hz)
1	171.1		172.7^b^		171.7	
2	131.9		134.1^b^		131.3	
3	136.6	6.70, t (7.6)	135.3^b^	6.64, t (7.0)	139.0	7.14, t (6.8)
4	28.8	2.76, dd (15.9, 7.6)	28.7	2.74, dd (15.9, 7.0)	35.4	2.70, dd (15.1, 6.8)
		2.82, dd (15.9, 7.6)		2.81, dd (15.9, 7.0)		2.91, dd (15.1, 6.8)
5	61.2,		61.3		74.2	
6	61.1	3.76, d (2.8)	61.1	3.76, d (2.7)	74.0	3.81, d (2.3)
7	65.9	4.83, br s	65.9	4.82, br s	72.5	4.27, d (2.3)
8	150.8		151.0		132.7	
9	131.9		131.8		135.1	
10	196.0		196.1		72.4	4.09, s
11	122.9	6.16, d (15.9)	123.6	6.15 d (16.0)	127.2	6.49, d (15.8)
12	139.9	5.86, dt (15.9, 7.0)	139.1	5.83, dt (16.0, 7.0)	135.2	6.04, dt (15.8, 7.0)
13	34.5	2.19, m	33.7	2.17, m	34.6	2.20, m
14	26.4	1.52, m	24.1	1.69, m	30.2	1.47, m
15	39.6	1.47, m	43.4	2.53, t (7.2)	32.6	1.34, m
16	68.4	3.73, q (6.2)	211.8		23.6	1.35, m
17	23.5	1.15, d (6.2)	29.9	2.14, s	14.4	0.92, t (7.0)
18	60.3	4.41, d (12.9) 4.52, d (12.9)	60.3	4.41, d (12.9) 4.51, d (12.9)	59.9	4.29, d (12.2) 4.46, d (12.2)
19	12.8	1.87, s	12.9^b^	1.87, s	12.9	1.91, s

^a^1H-NMR was recorded at 500 MHz; ^13^C-NMR was recorded at 125 MHz.

^b^Extracted from HSQC, HMBC data.

**Table 2:** ^1^H and 13C NMR spectroscopic data of compounds 4 and 5 in CD_3_OD^a^

No.	4		5	
	*δ*_C_	*δ*_H_ (mult., *J* in Hz)	*δ*_C_	*δ*_H_ (mult., *J* in Hz)
1	171.3		171.3	
2	132.0		131.9	
3	137.1	6.85, t (7.5)	137.2	6.86, t (7.5)
4	31.7	2.62, dd (15.8, 7.5)	31.8	2.63, dd (15.8, 7.5)
		3.01, dd (15.8, 7.5)		3.01, dd (15.8, 7.5)
5	62.1		62.2	
6	60.7	3.35, br s	60.8	3.35, br s
7	67.2	4.64, br s	67.5	4.68, br s
8	128.3		132.4	
9	136.6		133.9	
10	67.0	4.48, s	67.0	4.48, s
11	126.9	6.32, d (15.8)	127.0	6.40, d (15.8)
12	135.8	6.01, dt (15.8, 7.0)	134.8	5.99, dt (15.8, 7.0)
13	34.4	2.17, m	34.5	2.17, m
14	30.0	1.45, m	30.2	1.45, m
15	32.5	1.33, m	32.6	1.33, m
16	23.6	1.33, m	23.6	1.34, m
17	14.4	0.91, t (6.9)	14.4	0.91, t (6.9)
18	60.7	4.86^b^	58.2	4.35, s
19	12.8	1.87, s	12.8	1.87, s
20	172.9			
21	20.8	2.03, s		

^a^1H-NMR was recorded at 500 MHz; ^13^C-NMR was recorded at 125 MHz.

^b^Signal overlapped with water.

**Table 3:** NO inhibitory activity of compounds 1, 4–8 (IC_50_, μM)

Compounds	NO inhibitory Activity^a^
**1**	>100
**4**	88.66 ± 6.74
**5**	>100
**6**	11.20 ± 0.79
**7**	20.80 ± 1.41
**8**	>100
Positive control	Hydrocortisone
	53.68 ± 3.86

^a^The growth of RAW cells were not influenced by tested compounds at the concentration of 100 μM.
