# Supplementary material for: New ambuic acid derivatives from the solid culture of Pestalotiopsis neglecta and their nitric oxide inhibitory activity
Source: Sci Rep. 2015 May 19;5:9958. doi: 10.1038/srep09958 (PMC4437027; doi:10.1038/srep09958)
Supplement: Supplementary Information [file srep09958-s1.doc]

**Supplementary Information**

New ambuic acid derivatives from the solid culture of *Pestalotiopsis neglecta* and their nitric oxide inhibitory activity

Qiu-Yue Qi^1, 2, #^, Er-Wei Li^1, #^, Jun-Jie Han^1^, Yun-Fei Pei^1^, Ke Ma^1^, Li Bao^1, *^ Ying Huang^3^, Feng Zhao^4^, and Hong-Wei Liu^1, *^

^1^ State Key Laboratory of Mycology, Institute of Microbiology, Chinese Academy of Sciences, NO. 1 Beichen West Road, Chaoyang District, Beijing 100101, People's Republic of China

^2^ University of Chinese Academy of Sciences, No.19A Yuquan Road, Beijing 100049, People's Republic of China

^3^ State Key Laboratory of Microbial Resources, Institute of Microbiology, Chinese Academy of Sciences, NO. 1 Beichen West Road, Chaoyang District, Beijing 100101, People's Republic of China

^4^ School of Pharmacy, Yantai University, No. 32 Qingquan Road, Laishan District, Yantai, 264005, People's Republic of China

^#^ Authors contributing equally to this work.

* Authors to whom correspondence should be addressed;

E-Mail: [liuhw@im.ac.cn](mailto:liuhw@im.ac.cn) (H.W.L.); Tel: +86 10 64806074; fax: +86 10 64807515 and [baol@im.ac.cn](mailto:baol@im.ac.cn) (L.B.); Tel: +86 10 64806076; fax: +86 10 64807515.

# Contents

Figure S1 ^1^H NMR spectrum of compound **1** (500 MHz, CD_3_OD)

Figure S2 ^13^C NMR spectrum of compound **1** (500 MHz, CD_3_OD)

Figure S3 CD spectrum of compound **1** in methanol

Figure S4 ^1^H NMR spectrum of compound **2** (500 MHz, CD_3_OD)

Figure S5 ^13^C NMR spectrum of compound **2** (500 MHz, CD_3_OD)

Figure S6 CD spectrum of compound **2** in methanol

Figure S7 ^1^H NMR spectrum of compound 3 (500 MHz, CD_3_OD)

Figure S8 ^13^C NMR spectrum of compound **3** (500 MHz, CD_3_OD)

Figure S9 CD spectrum of compound **3** in methanol

Figure S10 The optimized conformers for **9a** in MMFF94

Figure S11 ^1^H NMR spectrum of compound **4** (500 MHz, CD_3_OD)

Figure S12 ^13^C NMR spectrum of compound **4** (500 MHz, CD_3_OD)

Figure S13 CD spectrum of compound **4** in methanol

Figure S14 ^1^H NMR spectrum of compound **5** (500 MHz, CD_3_OD)

Figure S15 ^13^C NMR spectrum of compound **5** (500 MHz, CD_3_OD)

Figure S16 CD spectrum of compound **5** in methanol


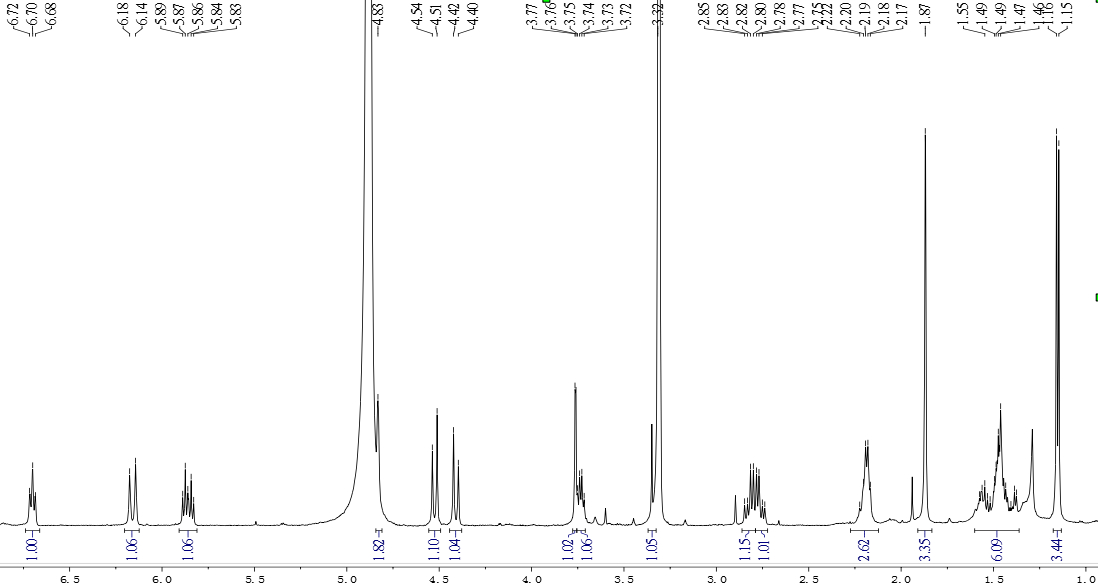


Figure S1 ^1^H NMR spectrum of compound **1** (500 MHz, CD_3_OD)


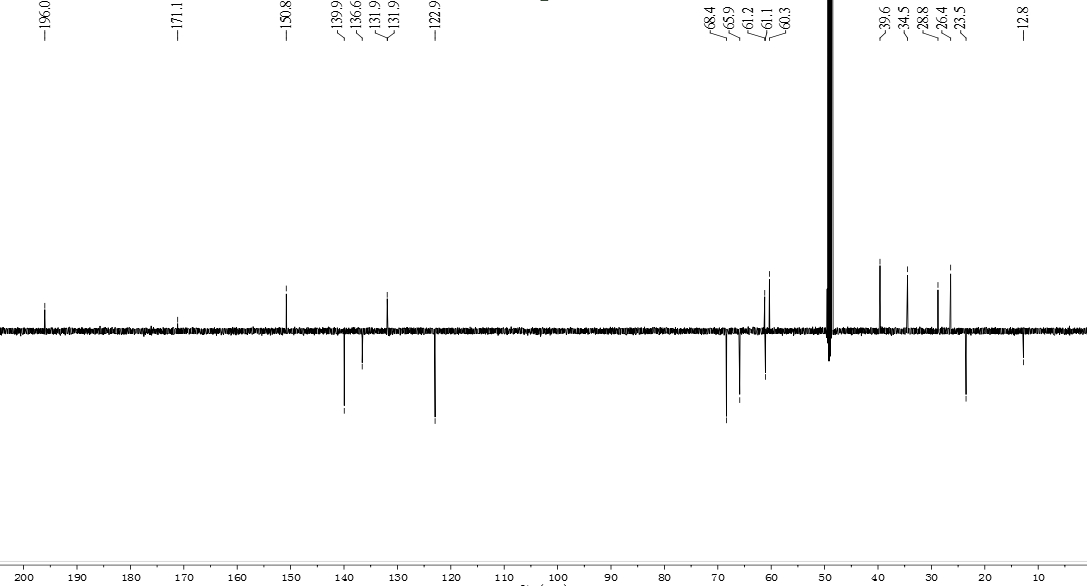


Figure S2 ^13^C NMR spectrum of compound **1** (500 MHz, CD_3_OD)


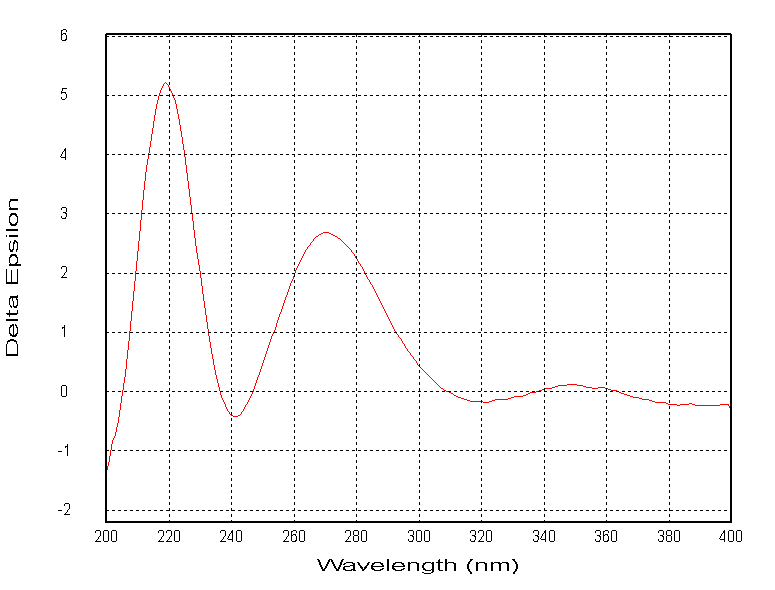


Figure S3 CD spectrum of compound **1** in methanol


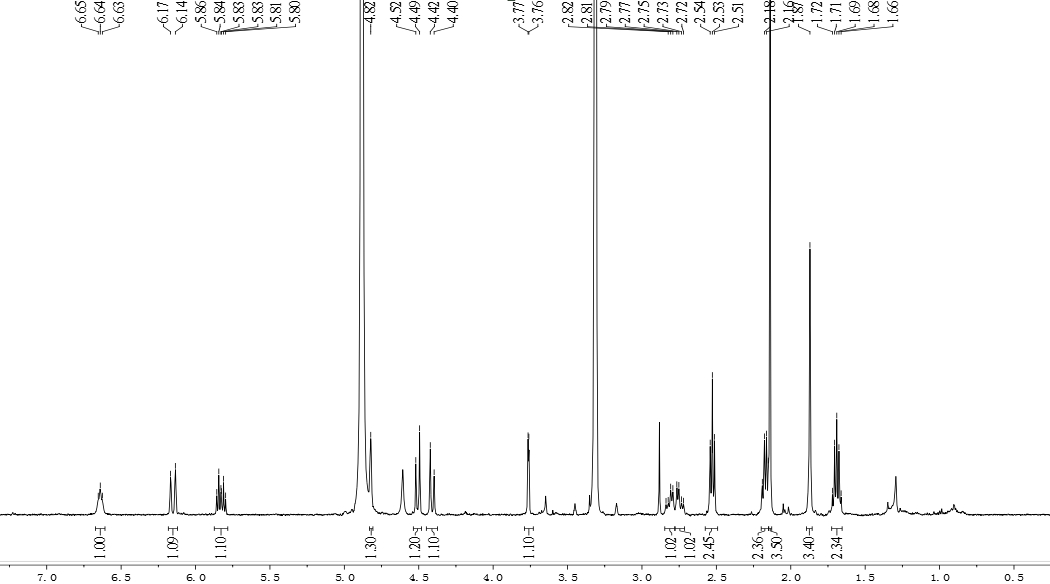


Figure S4 ^1^H NMR spectrum of compound **2** (500 MHz, CD_3_OD)


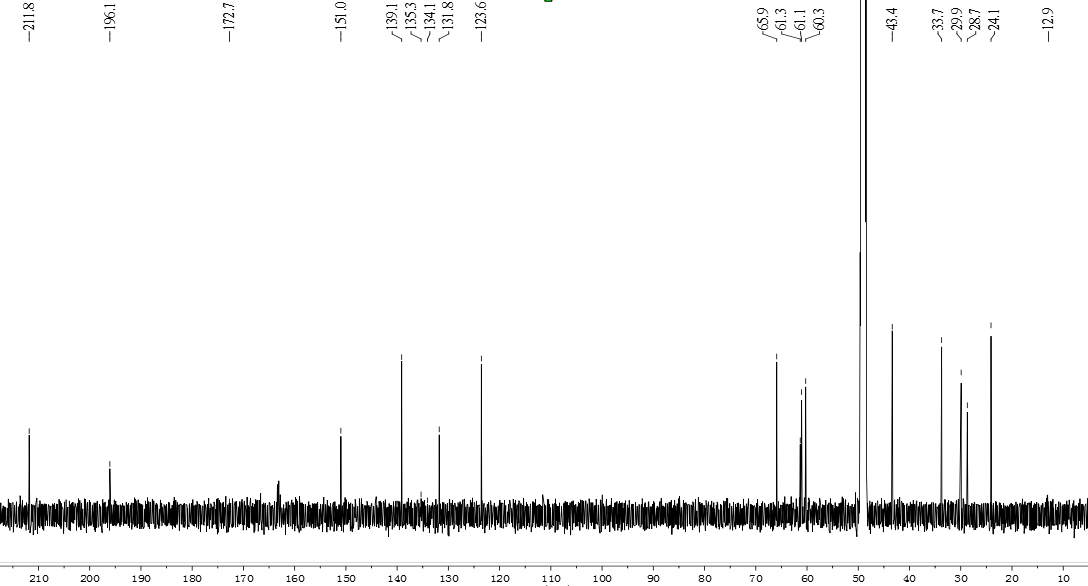


Figure S5 ^13^C NMR spectrum of compound **2** (500 MHz, CD_3_OD)


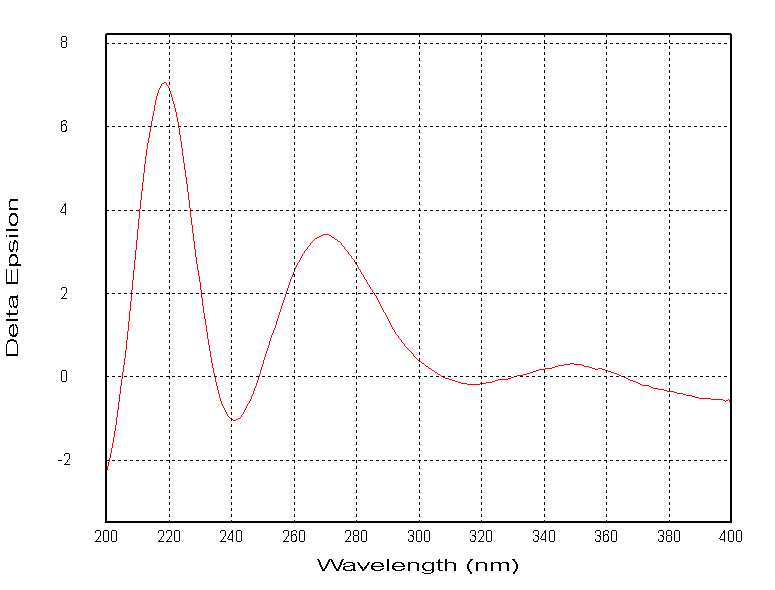


Figure S6 CD spectrum of compound **2** in methanol


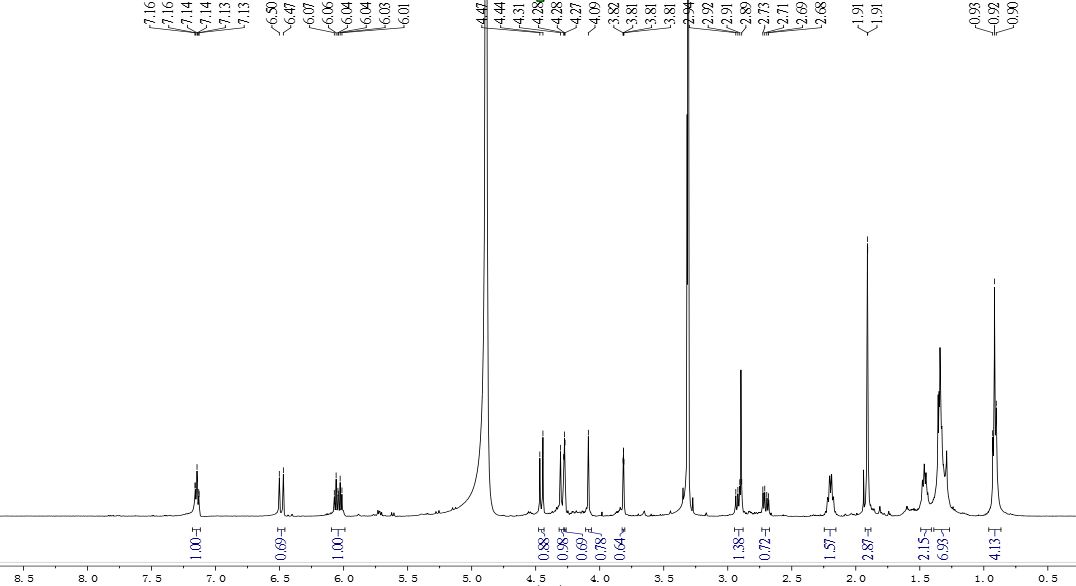
Figure S7 ^1^H NMR spectrum of compound **3** (500 MHz, CD_3_OD)


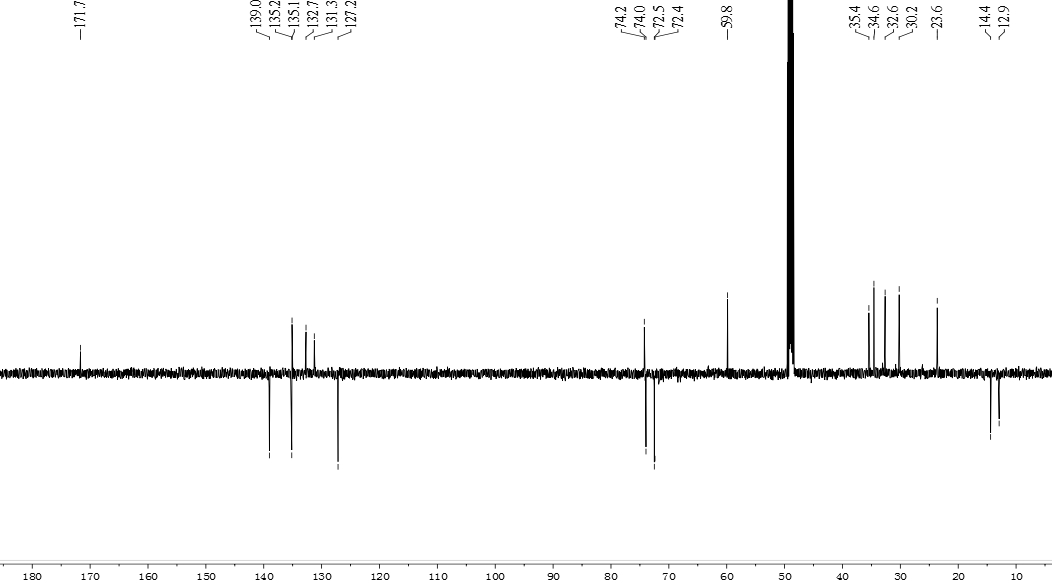


Figure S8 ^13^C NMR spectrum of compound **3** (500 MHz, CD_3_OD)


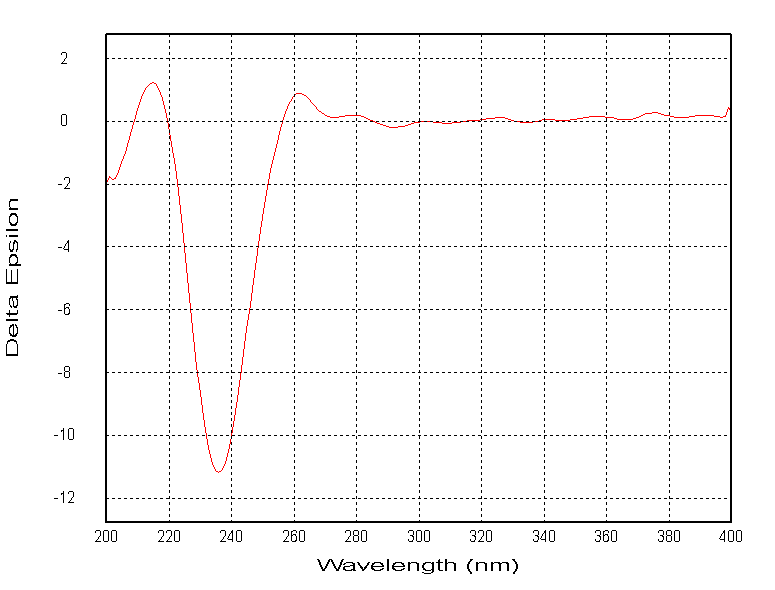


Figure S9 CD spectrum of compound **3** in methanol

33.6% 24.8% 29.6% 12.0%

Figure S10 The optimized conformers for **9a** in MMFF94


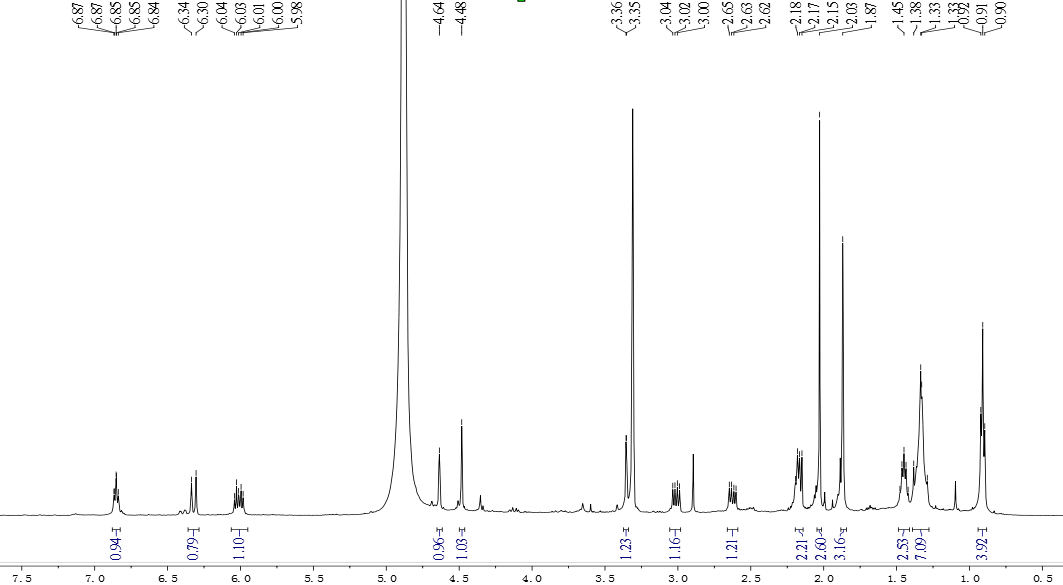


Figure S11 ^1^H NMR spectrum of compound **4** (500 MHz, CD_3_OD)


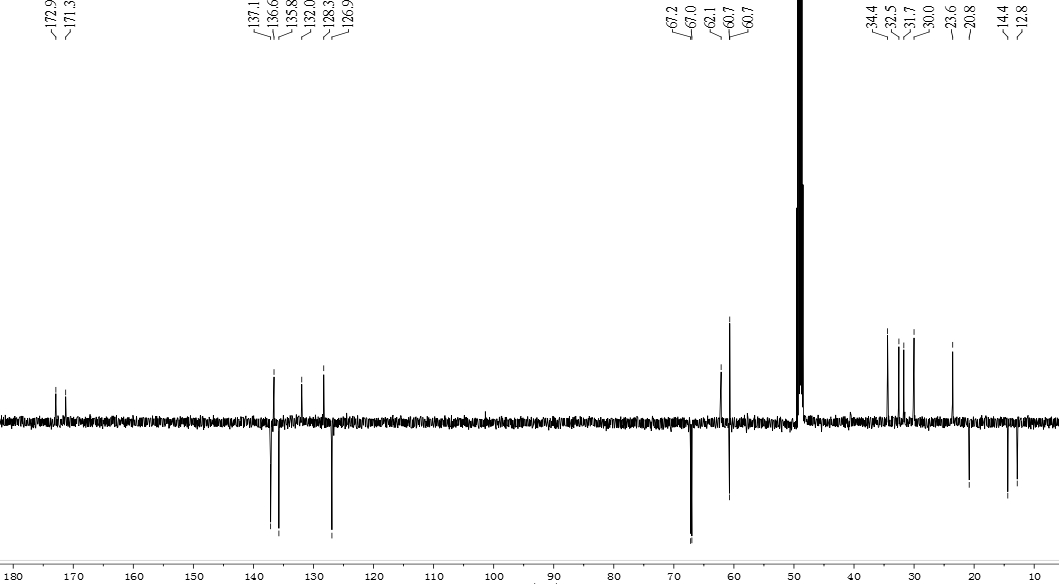


Figure S12 ^13^C NMR spectrum of compound **4** (500 MHz, CD_3_OD)


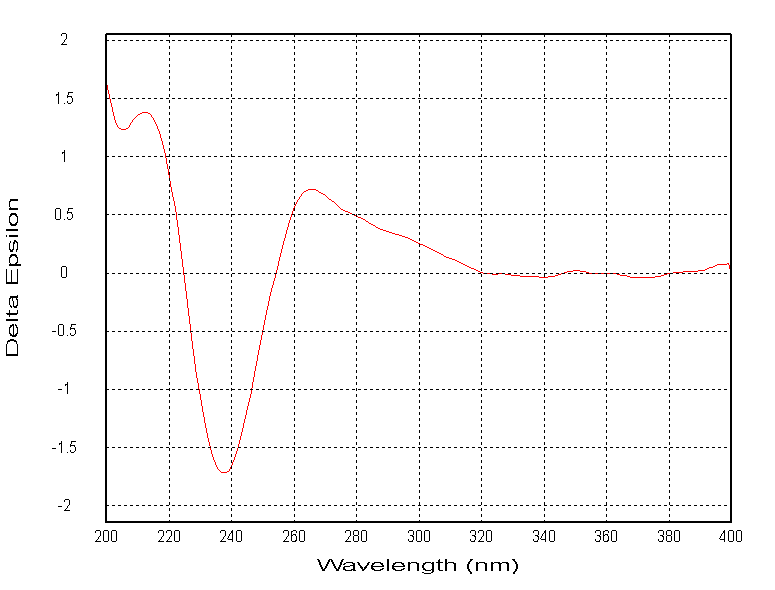


Figure S13 CD spectrum of compound **4** in methanol


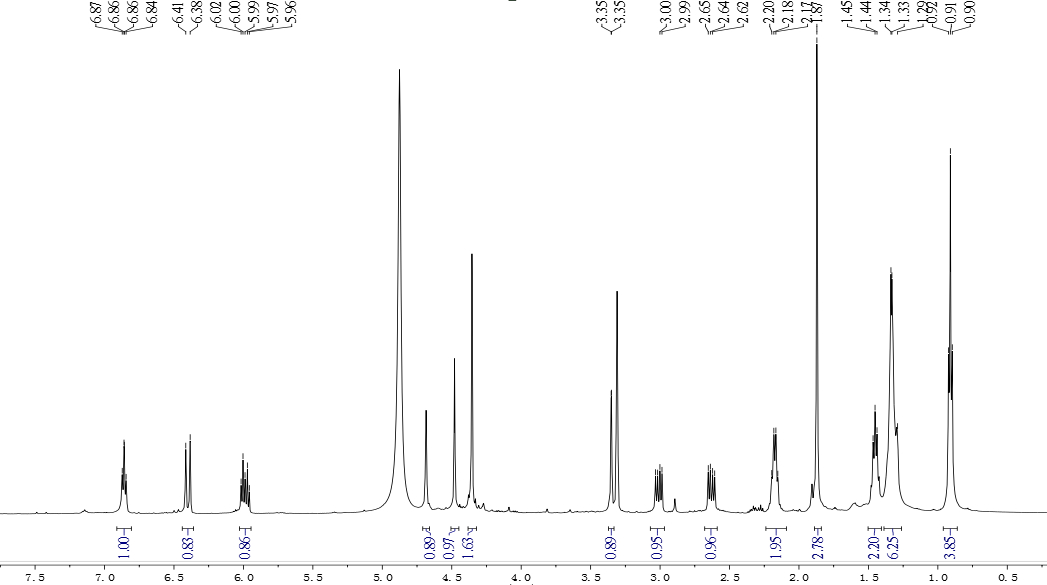


Figure S14 ^1^H NMR spectrum of compound **5** (500 MHz, CD_3_OD)


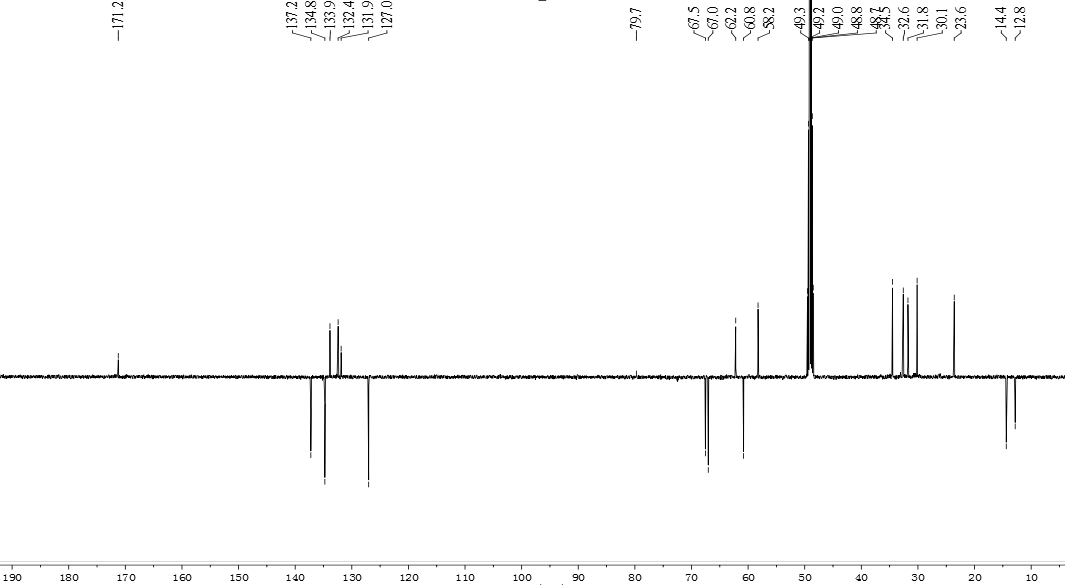


Figure S15 ^13^C NMR spectrum of compound **5** (500 MHz, CD_3_OD)


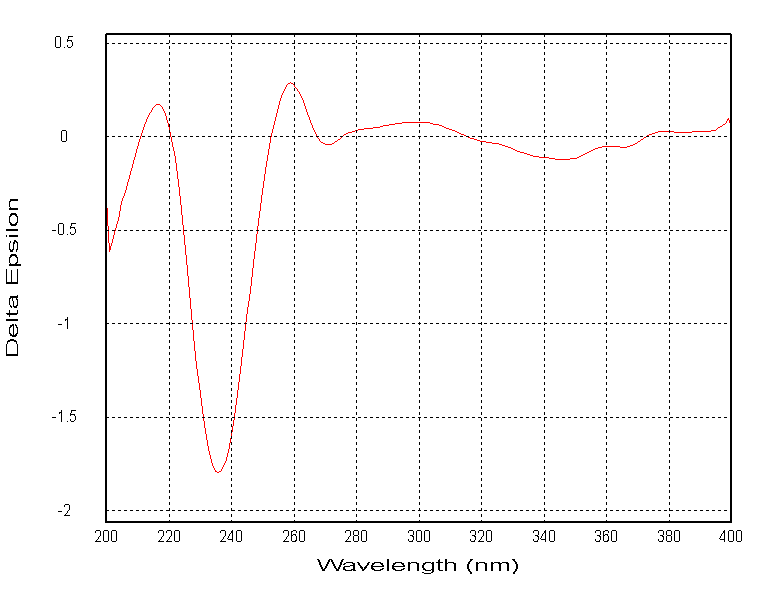


Figure S16 CD spectrum of compound **5** in methanol
